# Data Mining of Gene Arrays for Biomarkers of Survival in Ovarian Cancer

**DOI:** 10.3390/microarrays4030324

**Published:** 2015-07-17

**Authors:** Clare Coveney, David J. Boocock, Robert C. Rees, Suha Deen, Graham R. Ball

**Affiliations:** 1 John van Geest Cancer Research Centre, Nottingham Trent University, Nottingham NG11 8NS, UK; E-Mails: clare.coveney@ntu.ac.uk (C.C.); david.boocock@ntu.ac.uk (D.J.B.); robert.rees@ntu.ac.uk (R.C.R.); 2 Department of Histopathology, Queens Medical Centre, Derby Road, Nottingham, Nottinghamshire NG7 2NH, UK; E-Mail: suha.deen@nuh.nhs.uk

**Keywords:** ovarian cancer, meta-analysis, artificial neural networks, survival analysis, biomarkers, transcriptomics

## Abstract

The expected five-year survival rate from a stage III ovarian cancer diagnosis is a mere 22%; this applies to the 7000 new cases diagnosed yearly in the UK. Stratification of patients with this heterogeneous disease, based on active molecular pathways, would aid a targeted treatment improving the prognosis for many cases. While hundreds of genes have been associated with ovarian cancer, few have yet been verified by peer research for clinical significance. Here, a meta-analysis approach was applied to two carefully selected gene expression microarray datasets. Artificial neural networks, Cox univariate survival analyses and *T*-tests identified genes whose expression was consistently and significantly associated with patient survival. The rigor of this experimental design increases confidence in the genes found to be of interest. A list of 56 genes were distilled from a potential 37,000 to be significantly related to survival in both datasets with a FDR of 1.39859 × 10^−11^, the identities of which both verify genes already implicated with this disease and provide novel genes and pathways to pursue. Further investigation and validation of these may lead to clinical insights and have potential to predict a patient’s response to treatment or be used as a novel target for therapy.

## 1. Introduction

Ovarian cancer is the fifth most common cancer and the fourth most common cause of cancer related deaths in UK women. Each year approximately 7000 UK women are diagnosed with ovarian cancer and over 4000 succumb to the disease.

Ovarian cancer’s high mortality is attributed to the majority of incidences being diagnosed at a late stage. Few, if any, symptoms are expected from early stage disease, while in the later stages the indications are at most vague and more commonly attributed to non-pathological complaints including, back and abdominal pain, bloating and abnormal menstrual patterns [1].

Stage I ovarian cancer has a relatively good prognosis with 92% five-year survival, which drops down to 22% in patients with stage III disease. Despite the rising interest in identifying targeted therapy, there has not been significant change in disease outcome in the last few decades [2,3]. Currently, there is no screening tool with a performance specific or accurate enough to be implemented on the general population. Alongside ultrasonography, the existing tests for detection and monitoring of cancer progression or recurrence is based on serological immunoassay of Cancer Antigen 125 (CA125) [4,5]. This test is flawed by the natural variation and fluctuations of the protein [5,6,7], often false negative results lead to late presentation and diagnosis, and false positives to unnecessary explorative surgery [4]. However, encouragingly a recent report demonstrates the sensitivity of using CA125 as a screening tool for the general population to be vastly improved by using mathematical modeling to calculate risk based on serial measurements of CA125 [8].

Despite the continuing extensive study of ovarian cancer cell lines and patient material with numerous publications implicating novel genes associated with its incidence [9], little has changed in the treatment and expected outcome of patients presenting with ovarian cancer. Treatment for ovarian cancer is mainly total abdominal hysterectomy with bilateral salpingo-oophorectomy, omentectomy and staging. In advanced stage disease platinum based chemotherapy with or without taxol may be indicated as adjuvant or neoadjuvant therapy with interval debulking. Recently bevacizumab, an antiangiogenic therapy, has been used in certain cases [10,11]. A response to which is seen in approximately 70% of patients, however the majority of which develop a resistance to the therapy and experience a recurrence of the tumor, some more aggressively than others [10].

From the above, it is clear that there is an urgent need to identify non-invasive screening tools for early detection of ovarian cancer and also to improve targeted therapy for advanced stage disease.

DNA microarray experiments allow determination of the expression of entire genomes in DNA and RNA extracted from biological samples. To obtain the data in the current study, genetic material acquired from ovarian tumors was hybridized against a microarray gene chip containing probes for most of the characterized genes in the human genome yielding a relative expression value for several probes per gene [12]. These large, multidimensional, data could be interpreted using infinite analytical strategies to draw different conclusions [13]. Out of the thousands investigated and implicated genetic variants that are reported to have a role in ovarian cancer, only a few, have been exclusively positively replicated [9]. A recent review highlights agreement that instead of generating new experimental data, which can be both costly and timely, the sharing of resources, data, results, methods and samples is crucial to narrowing down common active cellular mechanisms in what is a relatively rare yet genotypically diverse disease [11].

The two methods of analysis explored in the current study are artificial neural networks (ANNs) and Cox proportional hazard modeling analysis. ANNs are a form of machine learning that are applied to non-linear datasets, pattern recognition algorithms to strengthen connections within its structure, which is akin to the plasticity of nervous systems in biology [14]. Cox proportional hazard modeling analysis is used to determine if a continuous independent variable such as gene expression levels associate with survival [15].

The two key focal points of research into ovarian cancer are firstly the development of a biomarker from a non-invasive test that can be used as a screening tool for early detection in the at risk population, and secondly to improve the prognosis and treatment of patients diagnosed with later stage disease.

The aim of the current study was to characterize genomic differences between tumors from patients that experienced different survival times after diagnosis with stage III ovarian cancer.

## 2. Experimental Section

**Figure 1:**
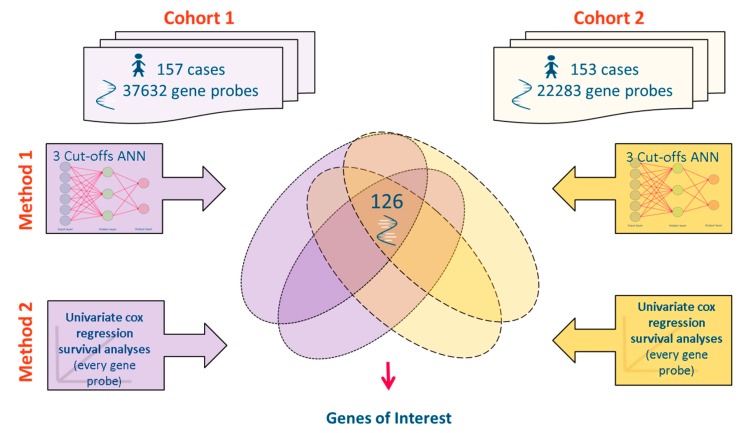
Two datasets (Cohort 1 containing 157 cases and 37,632 gene probes, Cohort 2 containing 153 cases and 22,283 gene probes) were mined for gene expression values significantly associating with ovarian cancer survival using two statistical approaches. Method 1: a set of three artificial neural networks (ANNs) using differing time point cut offs to define short and long term survival, Method 2; a Cox univariate survival analysis performed on every gene. Upon cross comparison of statistically interesting genes 126 gene probes were selected from a potential 37,632 for further analysis.

Figure 1 is a schematic depicting the meta-analysis approach used to filter two cohorts of data for genes that consistently significantly associate with patient survival time when analyzed using two cohorts of data and two analytical approaches.

### 2.1. Source Data

Array Express was searched for datasets comprising gene microarray data collected from cohorts of ovarian cancer samples with as similar profile as possible. Extraneous variables were minimized by searching Array Express and not including data acquired from experiments that did not fit a strict criteria: *i.e.*, including only data from large patient cohorts using micro-arrays representing the full genome. Datasets with low sample numbers, ambiguous or unclear sample data, studies based around cell lines, or with a focus on drug trials, were not included.

Survival time was the only dependent variable available in both the cohorts selected for the analysis. Patients in both studies selected were subject to the same treatment of possible debulking surgery, followed by platinum based chemotherapy [16,17].

### 2.2. Datasets Used

Gene array data were downloaded from Array Express, the dataset was built from tissue from patients with ovarian cancer who have been treated with the same care pathway. Full data and information is available at Array Express under experiments E-GEOD-13876 and E-GEOD-26712 [12].

Based on the patient information and data annotations provided with both datasets, survival time was selected as the basis for this investigation, *i.e.*, survival time was the only listed variable common to both data sets. Both of these datasets could be used to identify genes whose expression significantly and consistently associate with survival time from Stage III serous ovarian cancer, and, to validate or refute any genes recently reported to be linked to ovarian cancer but not fully validated.

Cohort 1:

Full data and information is available at Array express under the E-GEOD-13876 [12] Array: A-GEOD-7759-Operon human v3 ~35 K 70-mer two-color oligonucleotide microarrays. Sample information: 157 consecutive patients donated tumor from cyto-reductive surgery prior to platinum based chemotherapy treated at University Medical Center Groningen (UMCG, Groningen, The Netherlands) in the period 1990–2003 [17].

Cohort 2:

Full data and information is available at Array Express under experiment E-GEOD-26712 [12] Array: A-AFFY-33-Affymetrix GeneChip Human Genome HG-U133A [HG-U133A]. Sample information: 185 late-stage (III–IV) high-grade (2,3) ovarian cancer tumors donated from previously untreated patient at Memorial Sloan-Kettering Cancer Center between 1990 and 2003 [16].

### 2.3. Meta-Analysis of Microarray Data

A set of six three-layered back propagation ANNs with an architecture of 1 input node, 2 hidden layer nodes and 1 output node were trained to identify gene probes that perform well as predictors of short and long survival. The ANN algorithm was developed at NTU [14,18], contact CompanDX [19] for further details. Multiple ANNs were trained to accommodate a categorical analysis around a continuous variable. A backpropagation algorithm was used to update the weights of the ANN and was trained to convergence on an early stopping randomly extracted dataset comprising 20% of the global dataset. A sigmoidal transfer function was used in the architecture to relate input gene expression to survival. Firstly, the survival distribution of the population of the two datasets were observed, three possible cut-off time points determining short and long survival were defined; above and below 16, 23 and 30 months. Using these three survival cut-offs, ANN analyses were conducted on the two datasets. Within each of the six ANN analyses, the gene probes were ranked by their root mean gained error on an internal blind validation step comprising a different 20% of the global dataset and gene probes ranking below 0.05% were disregarded. The gene short names of these shortlisted gene probes were then cross-referenced across the three ANN from each time point in each dataset. Gene names were then weighted based on the frequency of their presence in the three ANNs top 0.05% ranking probes. The list of weighted gene names with a consistent predictive performance between long and short term survival were taken forward to the meta-analysis (see supplementary data for full gene probe listings).

Cox univariate survival analysis was conducted on every gene probe individually to determine the expression significantly correlated with survival. To do this, a macro was created within Statistica software that cycled round each of the thousands of gene probes within each dataset and produced a report for each one. Due to software limitations, this had to be done in several batches of 4000 probes for each dataset. The individual output reports were compiled and converted to an Excel spreadsheet. Gene probes were ranked by their *p*-value and any below 0.05 were disregarded. The gene codes of the gene probes with a *p*-value of ≤0.05 were taken forward for the meta-analysis (*p*-values available in supplementary data).

The Pivot table function within Excel was used to cross-compare the gene codes that performed well as predictors in the MLP-ANNs and had a significant *p*-value in the Cox univariate survival analysis. Gene probes that did not occur in all four categories were disregarded. The data corresponding to the gene probes of the genes identified to be of interest were extracted from the data. *T*-tests were conducted using the same time point cut-offs as described for the ANNs. Genes that did not have a significant *p*-value for one or more probe in both datasets were disregarded. Finally the mean averages of each were compared. Genes whose expression trends differed when correlated with survival between the datasets were disregarded.

The final list of 56 gene codes (Table 1) were cross-referenced using STRING to highlight any known association or link between them [20,21]. Literature and online resources such as Gene Cards and Human Protein Atlas were further mined to create a database of genomic, proteomic, expression, oncologic and pathway information to direct avenues of further investigation [22,23].

The probability this discovery occurring by chance was a probability of 1.39859 × 10^−11^. The number of genes found to be of interest multiplied by number of possible probes in each data set for both analyses ((56/37,632) × (56/22,283) × (56/37,632) × (56/22,283)) = 1.39859 × 10^−11^. If the work of Fury *et al.* [24] is taken into consideration, this probability may be even lower.

**Table 1:** Genes of Interest. The genes in the table above were found to significantly associate with survival time from stage III ovarian cancer.

Gene Code	Gene Name	Rank Order of Interest
DCN	decorin	1
EDNRA	endothelin receptor type A	2
GLT8D2	glycosyltransferase 8 domain containing 2	3
IGF2	insulin-like growth factor 2 (somatomedin A)///INS-IGF2 readthrough	4
MFAP4	microfibrillar-associated protein 4	5
PDZRN3	PDZ domain containing ring finger 3	6
PKD2	polycystic kidney disease 2 (autosomal dominant)	7
SEMA3C	sema domain, immunoglobulin domain (Ig), short basic domain, secreted, (semaphorin) 3C	8
IGFBP6	insulin-like growth factor binding protein 6	9
LDB2	LIM domain binding 2	10
NAV3	neuron navigator 3	11
NDN	necdin homolog (mouse)	12
OLFML3	olfactomedin-like 3	13
PCDH17	protocadherin 17	14
PJA2	praja ring finger 2, E3 ubiquitin protein ligase	15
PPFIBP1	PTPRF interacting protein, binding protein 1 (liprin β 1)	16
RARRES2	retinoic acid receptor responder (tazarotene induced) 2	17
SFRP4	secreted frizzled-related protein 4	18
BMP4	bone morphogenetic protein 4	19
HNRPDL	heterogeneous nuclear ribonucleoprotein D-like	20
LRRC17	leucine rich repeat containing 17	21
MAP4K4	mitogen-activated protein kinase kinase kinase kinase 4	22
PPP3CA	protein phosphatase 3, catalytic subunit, α isozyme	23
COLEC12	collectin sub-family member 12	24
IGFBP3	insulin-like growth factor binding protein 3	25
TNFAIP6	tumor necrosis factor, α-induced protein 6	26
BACH1	BTB and CNC homology 1, basic leucine zipper transcription factor 1	27
INTS5	integrator complex subunit 5	28
TNFRSF14	tumor necrosis factor receptor superfamily, member 14	29
ZFHX4	zinc finger homeobox 4	30
EFNB3	ephrin-B3	31
FYN	FYN oncogene related to SRC, FGR, YES	32
FZD7	frizzled family receptor 7	33
SCAMP1	secretory carrier membrane protein 1	34
TMEM45A	transmembrane protein 45A	35
NCOR1	nuclear receptor corepressor 1	36
BACH2	BTB and CNC homology 1, basic leucine zipper transcription factor 2	37
HIST1H3A	histone cluster 1, H3a	38
CLIP3	CAP-GLY domain containing linker protein 3	39
GULP1	GULP, engulfment adaptor PTB domain containing 1	40
PTPRE	protein tyrosine phosphatase, receptor type, E	41
SPAG9	sperm associated antigen 9	42
SPCS3	signal peptidase complex subunit 3 homolog (*S. cerevisiae*)	43
CTBP2	C-terminal binding protein 2	44
CDC25B	cell division cycle 25 homolog B (*S. pombe*)	45
GJB1	gap junction protein, β 1, 32 kDa	46
DCTD	dCMP deaminase	47
HBD	hemoglobin, delta	48
SLC11A2	solute carrier family 11 (proton-coupled divalent metal ion transporters), member 2	49
TPM2	tropomyosin 2 (β)	50
ZNF45	zinc finger protein 45	51
FHOD3	formin homology 2 domain containing 3	52
H2AFV	H2A histone family, member V	53
FKBP14	FK506 binding protein 14, 22 kDa	54
SMC3	structural maintenance of chromosomes 3	55
WTAP	Wilms tumor 1 associated protein	56

### 2.4. Verification of Protein Expression

From the literature and database mining, Endothelin receptor type A (EDNRA) was selected for verification at a protein level. A tissue MicroArray was purchased form Biomax (OV6161 from US Biomax Inc., Rockville, MD, USA [25]), and an Anti-EDNRA HPA014087 (Atlas Antibodies, Stockholm, Sweden) was selected above others for its demonstrated specificity via western blot of a human cell line. Biomax OV6161 is a high density microarray of 616 cores of paraffin-embedded ovarian specimens mounted onto a glass slide. It contains; 28 normal or normal adjacent tissue, 1 transitional cell carcinoma, 13 clear cell carcinoma and 280 cases of adenocarcinoma of varying stage and grade. All information is available at http://www.biomax.us/tissue-arrays/Ovary/OV6161 [25].

Slides were deparaffinized and dehydrated by heating at 60 °C on a hot plate for 10 min, immediately followed by two 5 min alcohol washes, and three 2 min washes in Industrial Methylated Spirits ending in ddH_2_O. Antigen retrieval consisted of a 20 min boil in a citrate buffer (pH6). After cooling in ddH_2_O, slides were carefully loaded to the Sequenza staining system and stained using the Novolink Polymer detection system (RE7200-CE, Leica Biosystems, Buckingham, UK) care was taken and checks were in place to ensure no part of the slide ever dried or microbubbles of air were trapped between the Sequenza coverslip and the slide, as per the manufactures recommendations. The dilution of the primary antibody was optimized using incomplete offcuts of a breast TMA and one additional test slide purchased from Biomax. A negative control omitting the primary antibody ensured all staining was associated with primary antibody binding. Two 5 min wash cycles rinsing with *tris*-buffered saline (TBS) were conducted between each of the following incubations; 5 min peroxidase block at room temperature to minimize non-specific binding, an 80 min room temperature incubation with the primary antibody HPA014087 (Atlas Antibodies, Stockholm, Sweden) at a 1 in 40 dilution. The antibody binding signal was amplified with a 30 min room temperature incubation with post primary reagent and a 5 min exposure to a 1 in 20 dilution of diaminobenzidine working solution. Finally, a 6 min incubation with the haematoxylin reagent enabled visualization of cell nucleic architecture. The stained slides were fixed by sequential alcohol washes in the reverse order they are listed above before sealing with a cover slip.

The TMA was accepted for scoring as a range of staining intensities were seen in tumor tissue across the slide. For a core to be considered viable to be scored, it had to contain at least 100 tumor cells. Cores were scored blindly on a categorical basis assigning a number to the overall intensity of the staining seen (0 negative, 1 weak, 2 moderate and 3 intense). Scores were assigned by a trained technician and a proportion (13.8%) were separately scored by a pathologist familiar with ovarian malignancies. The concordance between the scorers was very good (*κ* value = 0.921).

## 3. Results and Discussion

### 3.1. Genes of Interest

A list of 56 genes were distilled from a potential 37,000 gene probes to warrant further research into their role in survival time from ovarian cancer. These are listed in Table 1.

Completely different gene sets and numbers of genes in panels can be shown to be significantly differentially expressed between two datasets if different data mining methods are applied to the same data [26]. Of the final list of 56 genes of interest listed above, only three overlapped with those found to be of interest in the original publications. LRRC17 and TMEM45A were part of the panel of 86 genes found by continuous prediction algorithm to be of interest by Crijins *et al.* [17], GULP1 was also one of the 57 genes found to be of interest published by Bonome *et al.* [16]. The latter is intriguing as the paper’s primary analysis of fitting a Cox univariate survival curve to each gene is akin to Method 1 described above. This disparity can be attributed firstly to the stringency of using additional statistical analyses and validation of a second dataset as a filter to a genes significance, and secondly, the difference in data pre-processing and normalization strategies, which is known to alter the results to downstream analyses [17,26].

The rigor of combining a meta-analysis approach with multiple testing using a variety of statistical approaches, increases the power and confidence in the relevance of genes found to be of interest and ensures the probability of these findings to have occurred by chance to be infinitesimal; only the most “robust” biomarkers remained. Encouragingly, the 56 genes of interest included are both known and novel candidates associating with ovarian cancer survival. Namely, IGF2 is overexpressed in ovarian cancers, increased ligation is seen ovarian cystic fluid [27], which activates molecular pathways key to cell invasion [28], and, independently is a predictor of poor survival [29]. IGFBP3 and IGFBP6 are part of these pathways and the former is downstream of a p53 cascade. BMP4 is a known mediator of ovarian metastasis and cell invasion [30], its increased expression is a predictor of poor survival [31], and, has been implicated in cisplatin resistance [31]. Others such as WTAP, MAPK, and NAV3 have been implicated in other cancers but less so for ovarian [32,33,34].

This broad, meta-analytical approach benefits from being comprehensive; however, the loss of the ability to control extraneous variables is an inherent challenge when using publically sourced data. There are numerous non-recorded variables that could also determine patient survival times, this was and should always be acknowledged and considered when assumptions during the interpretation of results are made in order to hypothesize and derive possible meaning.

As both patient data cohorts received the same care pathway of primary debulking surgery followed by platinum based chemotherapy, chemoresistance will have been a contributing factor to survival times for a proportion of those patients. It could be suggested that the differential expression of at least some of the 56 genes of interest are a consequence of up or down-regulation of genes within tumors making them either more aggressive or to be able to evade platinum based chemotherapy. IGFBP3 has been shown to mediate resistance to cisplatin therapy in non-small-cell lung cancer [35], and BMP-4 expression has been shown to be altered after chemotherapy [31].

### 3.2. Preliminary Validation

Based on collated information from databases and literature review, EDNRA was selected as an interesting starting point to begin verification of genes protein expression patterns in relation to ovarian cancer: Epithelial to mesenchymal transition (EMT) was a common theme when collating information of the 56 genes of interest. Cell line studies have also implicated the phenomena of EMT to occur in platinum based drug resistance in epithelial ovarian cancer [36]. However, the exact mechanisms by which this happens are unconfirmed, in fact conflicting results are reported from both *in* *vivo* and *in* *vitro* studies [37]. The presence of markers of EMT such as SNAIL and E-cadherin have been linked with ovarian cancer invasiveness [36] and the activation of anti-apoptotic pathways such as NF-κB have been observed in cisplatin resistant cell lines [37]. Contrary to prior evidence, Miow *et al.* [37] found cisplatin had a higher efficacy on ovarian cell lines with mesenchymal status than those with an epithelial status.

Rosano *et al.* [36] elucidates EDNRA role in cell signaling pathways in the context of EMT in ovarian cancer cell line. An examination of EDNR2A expression in a wider cohort of ovarian specimens such as a tissue microarray would better represent the heterogeneity of ovarian cancers—hence its selection for this study.

**Figure 2:**
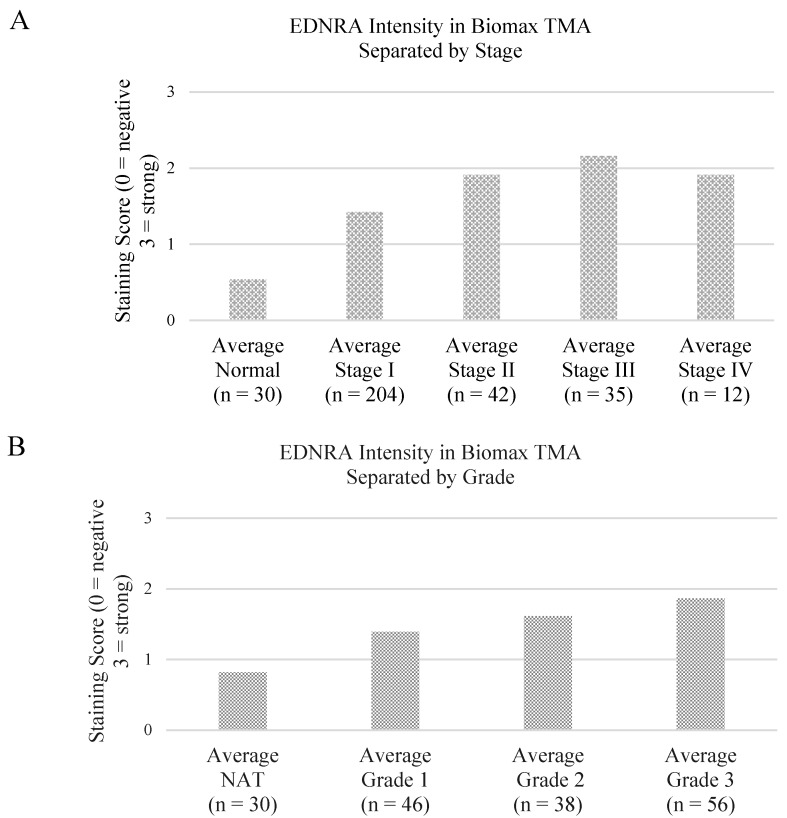
Endothelin receptor type A (EDNRA) Protein Expression in Ovarian Tissue of Different Stages and Grades. (**A**) A bar graph of protein expression score and cancer stage; (**B**) A bar graph of protein expression score and cancer grade.

A clear increase in EDNRA protein expression was seen in the higher grade and later stage disease (Figure 2, Table 2 and Table 3). Endothelin receptor type A (EDNRA) is the primary receptor for endothelin-1. Activation of EDNRA initiates G protein coupled receptor (GPCR) mediated activation of phosophatidylinositol-calcium second messenger system [13]. Its increased expression in the more intense cancers is likely representing increased cell proliferative activity of the tumors. A tissue microarray from a cohort of patients matching the profile of those in the microarray cohorts with survival data would expand upon this.

**Figure 3:**
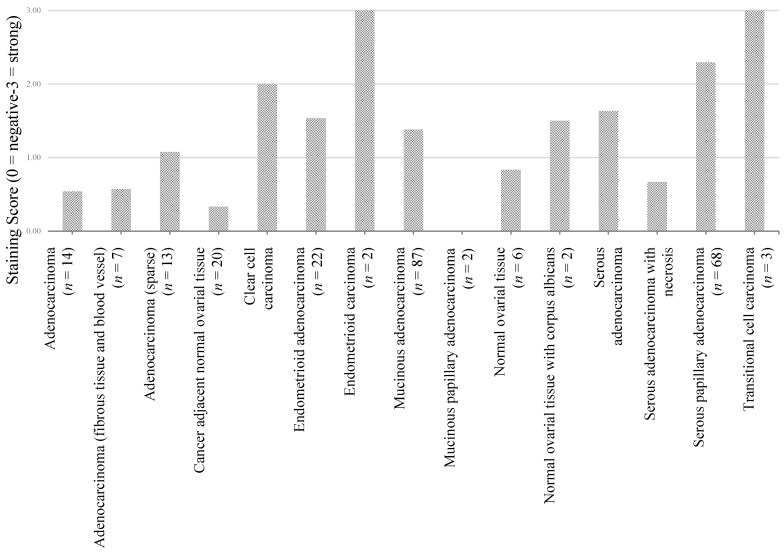
EDNRA protein expression in ovarian tumor histologies. A bar graph of protein expression score separated by disease histotypes.

Significantly differential staining was also seen in different types of ovarian tumor (Figure 3 and Table 4) implying that expression has potential to subgroup different histotypes of tumor. However there are insufficient numbers to draw any firm conclusions from these.

Further investigation and validation of the genes that have not yet been reported to associate with survival and investigating commonalities between the novel and known genes may have clinical relevance and have potential to predict a patient’s response to treatment or be used as a novel target for therapy.

Moreover, using the genes in combination with each other as a gene signature or biomarker panel and clarifying the nature of these commonalities using more, freely available online resources such as STRING, KEGG, Reactome, BioGrid, Panther and HeTop could begin to unearth molecular pathways with potential to characterize the nature of individual tumors within patient cohorts and enable more tailored treatment.

**Table 2:** *T*-test table comparing the significance of protein expression differences.

*p* value	Normal	Stage I	Stage II	Stage III	Stage IV
Normal	-	2.1974 × 10^−5^	1.00711 × 10^−8^	2.2073 × 10^−11^	9.99574 × 10^−7^
Stage I	-	-	0.000137099	8.5081 × 10^−8^	0.000137099
Stage II	-	-	-	0.15060521	0.998291248
Stage III	-	-	-	-	0.316994038
Stage IV	-	-	-	-	-

**Table 3:** *T*-test table comparing the significance of protein expression differences.

*p* value	All NAT	All Grade 1	All Grade 2	All Grade 3
All NAT	-	0.005302566	4.64816 × 10^−6^	1.36028 × 10^−10^
All Grade 1	-	-	0.244156689	0.007596408
All Grade 2	-	-	-	0.07998109
All Grade 3	-	-	-	-

**Table 4:** *T*-test *p*-values comparing EDNRA protein expression between cancer histology. Italicized numbers indicate *p*-value less than 0.05.

	Adenocarcinoma (*n* = 14)	Adenocarcinoma (fibrous tissue and blood vessel) (*n* = 7)	Adenocarcinoma (*n* = 13)	Cancer adjacent normal ovarial tissue (*n* = 20)	Clear cell carcinoma (*n* = 26)	Endometrioid adenocarcinoma (*n* = 22)	Endometrioid carcinoma (*n* = 2)	Mucinous adenocarcinoma (*n* = 87)	Mucinous papillary adenocarcinoma (*n* = 2)	Normal ovarial tissue (*n* = 6)	Normal ovarial tissue with corpus albicans (*n* = 2)	Serous adenocarcinoma (*n* = 339)	Serous adenocarcinoma ith necrosis (*n* = 6)	Serous papillary adenocarcinoma (*n* = 68)	Transitional cell carcinoma (*n* = 3)
Adenocarcinoma (*n* = 14)	-	0.91	0.18	0.37	*0.01*	0.06	*0.00*	0.07	0.19	0.35	0.08	*0.01*	0.81	*0.00*	*0.00*
Adenocarcinoma (fibrous tissue and blood vessel) (*n* = 7)	-	-	0.09	0.37	*0.00*	*0.01*	*0.00*	*0.01*	0.28	0.33	0.08	*0.00*	0.72	*0.00*	*0.00*
Adenocarcinoma (*n* = 13)	-	-	-	*0.01*	*0.02*	0.34	*0.01*	0.37	0.11	0.52	0.52	0.05	0.34	*0.00*	*0.01*
Cancer adjacent normal ovarian tissue (*n* = 20)	-	-	-	-	*0.00*	*0.00*	*0.00*	*0.00*	0.45	0.07	*0.02*	*0.00*	0.29	*0.00*	*0.00*
Clear cell carcinoma (*n* = 26)	-	-	-	-	-	0.09	0.28	*0.03*	*0.04*	*0.03*	0.59	0.09	*0.02*	0.23	0.28
Endometrioid adenocarcinoma (*n* = 22)	-	-	-	-	-	-	0.05	0.92	0.08	0.21	0.59	0.32	0.12	*0.00*	0.05
Endometrioid carcinoma (*n* = 2)	-	-	-	-	-	-	-	0.05	-	*0.00*	0.10	0.06	*0.01*	0.26	-
Mucinous adenocarcinoma(*n* = 87)	-	-	-	-	-	-	-	-	0.10	0.25	0.89	0.06	0.14	*0.00*	0.05
Mucinous papillary adenocarcinoma (*n* = 2)	-	-	-	-	-	-	-	-	-	*0.03*	0.10	*0.02*	0.32	*0.00*	-
Normal ovarian tissue (*n* = 6)	-	-	-	-	-	-	-	-	-	-	0.13	0.05	0.66	*0.00*	*0.00*
Normal ovarian tissue with corpus albicans (*n* = 2)	-	-	-	-	-	-	-	-	-	-	-	0.85	0.25	0.20	0.10
Serous adenocarcinoma (*n* = 339)	-	-	-	-	-	-	-	-	-	-	-	-	*0.02*	*0.00*	0.06
Serous adenocarcinoma with necrosis (*n* = 6)	-	-	-	-	-	-	-	-	-	-	-	-	-	*0.00*	*0.01*
Serous papillary Adenocarcinoma (*n* = 68)	-	-	-	-	-	-	-	-	-	-	-	-	-	-	0.26
Transitional cell Carcinoma (*n* = 3)	-	-	-	-	-	-	-	-	-	-	-	-	-	-	-

It should be emphasized that the reporting of each of these genes association with survival from ovarian cancer may not be novel, however the genes that emerge to appear alongside each other consistently over a number of experiments, technologies and cohorts will elucidate commonalities, signaling pathways and cell processes active that would lead to subcategorization of tumors. Unfortunately, it is likely that the results seen here, as in all multidimensional analyses of large cohorts are further corrupted by the heterogeneity of both the cases within the disease, and the cells within each tumor microenvironment. It is unlikely a disease as phenotypically diverse and poorly characterized as ovarian cancer will have one or a few subcategories. Multiple onco-genotypes and onco-phenotypes are likely to be present within any cohort dampening the potential for each to be discovered.

## 4. Conclusions

A list of 56 genes have been filtered from a meta-analysis of gene micro-array data. A proportion of these are well characterized in cancer, this both confirms the reliability of the methods and data used, and opens avenues of research to peruse to further our understanding of the genetics of the disease.

Validation at protein level was begun with the IHC of an ovarian TMA (322 ovarian specimens) for EDNRA. A significant association was seen between EDNRA expression and ovarian cancer stage and grade.

Future investigations EDNRA in ovarian tumors, where survival data is available, would elucidate its potential role identifying subpopulations of patients and direct treatment accordingly.

## Supplementary Materials

Supplementary materials can be found at http://www.mdpi.com/2076-3905/4/3/324/s1.
